# Filtering data from the collaborative initial glaucoma treatment study for improved identification of glaucoma progression

**DOI:** 10.1186/1472-6947-13-137

**Published:** 2013-12-21

**Authors:** Greggory J Schell, Mariel S Lavieri, Joshua D Stein, David C Musch

**Affiliations:** 1 Department of Industrial and Operations Engineering, University of Michigan, Ann Arbor, Michigan, USA; 2 Department of Ophthalmology and Visual Sciences, University of Michigan, Ann Arbor, Michigan, USA

## Abstract

**Background:**

Open-angle glaucoma (OAG) is a prevalent, degenerate ocular disease which can lead to blindness without proper clinical management. The tests used to assess disease progression are susceptible to process and measurement noise. The aim of this study was to develop a methodology which accounts for the inherent noise in the data and improve significant disease progression identification.

**Methods:**

Longitudinal observations from the Collaborative Initial Glaucoma Treatment Study (CIGTS) were used to parameterize and validate a Kalman filter model and logistic regression function. The Kalman filter estimates the true value of biomarkers associated with OAG and forecasts future values of these variables. We develop two logistic regression models via generalized estimating equations (GEE) for calculating the probability of experiencing significant OAG progression: one model based on the raw measurements from CIGTS and another model based on the Kalman filter estimates of the CIGTS data. Receiver operating characteristic (ROC) curves and associated area under the ROC curve (AUC) estimates are calculated using cross-fold validation.

**Results:**

The logistic regression model developed using Kalman filter estimates as data input achieves higher sensitivity and specificity than the model developed using raw measurements. The mean AUC for the Kalman filter-based model is 0.961 while the mean AUC for the raw measurements model is 0.889. Hence, using the probability function generated via Kalman filter estimates and GEE for logistic regression, we are able to more accurately classify patients and instances as experiencing significant OAG progression.

**Conclusion:**

A Kalman filter approach for estimating the true value of OAG biomarkers resulted in data input which improved the accuracy of a logistic regression classification model compared to a model using raw measurements as input. This methodology accounts for process and measurement noise to enable improved discrimination between progression and nonprogression in chronic diseases.

## Background

Open angle glaucoma (OAG) is a chronic degenerative ocular disease characterized by damage to the optic nerve, which when poorly managed can lead to blindness. Glaucoma is the second leading cause of blindness with an estimated 2.2 million adult Americans diagnosed with glaucoma [1,2]. Patients with OAG are monitored regularly via visual field (VF) tests and intraocular pressure (IOP) readings [3-8]. Clinicians use the results of these monitoring techniques to determine whether significant disease progression has occurred, i.e. a change in the patient’s disease characteristics which calls for changes in treatment decisions. However, these clinical observations are subject to process and measurement noise. Errors in machine calibration, patient anxiety, human error in administering tests, and variations in measurement technique can all contribute to measurement noise when assessing chronic diseases [9]. Biological variability, like intraday fluctuation of intraocular pressure, is a contributing factor to process noise which can affect the ability to identify significant disease progression [10]. Distinguishing between signal and noise becomes paramount when these noisy measurements are used in decision making. While most clinicians (glaucoma specialists in particular) are aware of the variability in VF and IOP measurements, non-specialists may not fully appreciate the importance of considering noise in VF findings and IOPs from visit to visit: they may erroneously conclude a patient is progressing or non progressing when they observe variability and make treatment decisions accordingly. Our proposed methodology provides clinicians with a mathematical framework that systematically accounts for noise in the data used to predict disease progression to aid clinicians (both specialists and non-specialists) in their decision making process.

In order to determine when a patient with OAG should be observed by his/her physician, a dynamic and personalized algorithm was developed using a Kalman filter approach to estimate VF test and IOP measures (Helm J, Lavieri M, Oyen MV, Stein J, Musch D: Dynamic forecasting and control algorithms of glaucoma progression for clinician decision support, unpublished). These estimates were then mapped to a probability of experiencing OAG progression via logistic regression to determine whether significant progression occurred that would signal the need for additional testing and/or impact treatment decisions. The development and the implications of using Kalman filter estimates in identifying disease progression are the focus of this work.

Kalman filtering is a technique for identifying signal in the presence of measurement and process noise. The Kalman filter approach has been used to estimate pulmonary blood flow [11], track cardiovascular signals [12], continuously monitor glucose levels [13], and monitor prostate specific antigen levels in prostate cancer patients [14]. These applications of the Kalman filter are used for predicting important health metrics, but the relationship between filtered estimates of these metrics and significant disease progression has not yet been modeled.

Identifying significant disease progression requires analysis of the longitudinal data of a particular patient. Therefore, we used generalized estimating equations (GEE) to statistically model the relationship between Kalman filter estimates of OAG health metrics and progression. GEE has been extensively used in the medical literature: to assess improvements from conversion to electronic health records [15], to identify risk factors for chronic obstructive pulmonary disease [16], to identify predictors of influenza vaccine acceptance [17], and to study spatially correlated binary data in neuroimaging [18]. However, applying GEE to raw measurements often leads to decision making that is informed by “noisy” observations, not measurements which reflect the true disease dynamics.

The aim of this work is to show that utilization of Kalman filter estimates of patient health metrics in logistic regression models improves significant disease progression identification compared to logistic regression models constructed using raw clinical observations. Furthermore, Kalman filter estimates explicitly account for process and measurement noise inherent in clinical data, making these estimates more informative than raw observations alone. This is of particular interest to clinicians who must decide when to monitor patients and select treatments based on the patient’s likelihood of progression. While our initial application is to patients with OAG, this methodology is applicable to other chronic diseases.

## Methods

Our proposed methodology combines an understanding of system dynamics properties through a Kalman filter approach with the marginal response technique of GEE to estimate the true value of clinical observations and to improve the ability of the logistic regression models to identify significant OAG progression. Our methodology is as follows: first, we built our dataset for analysis from clinical observations of a randomized clinical trial of OAG patients. Next, we constructed a robust definition of significant OAG progression based on the knowledge of subject matter experts. We then applied Kalman filtering to the repeated measures data of the large-scale randomized controlled clinical trial to estimate true values of variables believed to be correlated to significant OAG progression. We then applied GEE with a logit link function to the filtered data to develop a probability function for significant OAG progression. Finally, through cross validation, we measured sensitivity and specificity and calculated the area under the receiver operating characteristic (ROC) curve (AUC) to evaluate the accuracy of the probability function at identifying significant OAG progression.

All analyses were performed in R. Funding has been received by grant UL 1RR024986 from the National Institutes of Health (NIH) and grant 1161439 from the National Science Foundation (NSF).

All study centers involved in CIGTS obtained institutional review board approval for the study. University of Michigan institutional review board approval was granted for the continued analysis of the study results (HUM00037985).

### Data

The data set used for parameterization and validation of our proposed methodology came from the Collaborative Initial Glaucoma Treatment Study (CIGTS). CIGTS provided clinical visit data for 607 patients with early to moderate OAG over 10 years. All patients were seen approximately every 6 months following initial intervention to have a VF test and IOP check. Longitudinal mean deviation (MD) and pattern standard deviation (PSD) values from VF tests, along with longitudinal IOP measurements, were obtained for each patient from the clinical trial data. From the longitudinal measurements, we calculated velocities and accelerations for MD, PSD, and IOP for every patient at each visit. We also extracted demographic information (age, sex, and race) for every patient in the clinical trial.

Our inclusion criteria required patients to have at least 4 follow-up VF tests and IOP checks after initial intervention. We also required the patient’s VF test data to include information on each individual light sensitivity point in order to apply our progression definition. Further, patients were required to have been initially treated with medical therapy. Patients were censored when they left the clinical trial or if they underwent trabeculectomy or argon laser trabeculoplasty (ALT).

### Progression labeling

Our modeling approach used repeated measures data from a randomized clinical trial to obtain information about the evolution of OAG over time. Given the set of longitudinal data obtained sequentially for each patient over the course of the clinical trial, we used the input of subject matter experts to retrospectively identify patients that experienced a significant change in disease characteristics that would warrant clinical intervention. It is important to note that our definition of significant disease progression is meant to serve as an alert to clinicians. Clinicians use the information obtained from the models along with their experience and patient-specific factors to ultimately decree how to best care for a particular OAG patient.

A large drop in MD is generally accepted as an instance of OAG progression. Furthermore, we used the Hodapp-Anderson-Parish (HAP) classification [19] in our definition of significant OAG progression. Patients were labeled as experiencing progression at visit *j* when there was a loss of ≥3 decibels (dB) of MD with respect to baseline MD at visit *j* and this loss of MD also occured for some future visit *k* : *k* > *j* or if the patient shifted upward in HAP class (e.g. moderate to severe). We applied this definition to all patients in our dataset. This definition of progression requires both significant change in disease characteristics at the particular visit and a validation of this change in some future visit. Validation of the loss of MD at a future visit mitigates the chance of erroneously concluding a patient is progressing or not progressing due to noise in the data. In practice, this added level of validation necessitates the development of an OAG disease progression probability function, since knowledge about future visits is not available to clinicians when identifying whether a patient has progressed.

Our definition of OAG progression (using either a validated 3 dB decline in MD or worsening based on HAP criteria) was compared against other suggested definitions of OAG progression (validated decline of 3 dB in MD alone, progression based on HAP criteria alone, and a point-wise linear regression method for progression detection) [20] and the model performed well irrespective of the OAG progression definition chosen. Given our interest in trying to identify global worsening of visual field from glaucoma as well as segmental areas of the visual field loss from glaucoma, we opted to use the more encompassing progression definition, characterized as a validated decline in MD of 3 dB from baseline or worsening based on HAP criteria for our analyses.

### Application of Kalman filter

The raw measurements obtained from sequential testing of OAG patients can be susceptible to process and measurement noise. To mitigate the effect of process and measurement noise, we utilized a Kalman filter approach to estimate the true value for observations obtained from VF and IOP tests. The Kalman filter utilizes recursive mathematical equations to optimally estimate the mean and covariance parameters of a process to characterize the state of the disease system [21]. The disease state can be multidimensional. We considered our state, $\alpha_{i,j}\in R^{n}$, to be the value of OAG-related variables (MD, PSD, IOP, and their respective velocities and accelerations) at the *j*^*th*^ observation time for patient *i*. The Kalman filter assumes the state is a Gaussian random variable and that the evolution of the disease state is governed by a linear stochastic difference equation:

(1) $$\begin{array}{l} \alpha_{i,j}=T\alpha_{i,j-1}+w_{i,j} \end{array}$$

Equation (1) represents the disease state, *α*_*i*,*j*_, of patient *i* at the current period *j*, as a transformation of the state of the last period, *α*_*i*,*j*-1_, according to the transition matrix **T**, plus Gaussian process noise, *w*_*i*,*j*_, with mean 0. Additionally, the covariance of the disease state variables for the current period, *Σ*_*i*,*j*_, is also governed by the transition matrix **T**, plus the covariance matrix of the process noise, **Q**.

$$\begin{array}{l} \Sigma_{i,j}=T\Sigma_{i,j-1}T^{\prime }+\text{Q} \end{array}$$

Moreover, the true state cannot be directly measured. Instead, the clinical observations, *z*_*i*,*j*_, are assumed to be a linear function of the state, *α*_*i*,*j*_, transformed by the matrix **H** plus Gaussian measurement noise, *v*_*i*,*j*_, with mean 0:

$$\begin{array}{l} z_{i,j}=\text{H}\alpha_{i,j}+v_{i,j} \end{array}$$

As described by (Helm J, Lavieri M, Oyen MV, Stein J, Musch D: Dynamic forecasting and control algorithms of glaucoma progression for clinician decision support, unpublished), we used the expectation maximization (EM) algorithm for parameter estimation [22].

We obtained state estimates, ${\hat{\alpha }}_{i,j}$ for each patient’s visit by recursively predicting the disease state values using a population-based understanding of OAG mechanics (i.e. parameterized transition and covariance matrices) and updating the estimates to reflect the patient’s particular disease evolution [21]. First, Equation (1) is used to forecast the patient’s disease state in the next period. The Kalman filter then uses the patient’s observed disease state in order to update the estimate of the true state, ${\hat{\alpha }}_{i,j}$. This personalized trajectory process is repeated for each patient, over the course of the patient’s duration in the clinical trial, to obtain the best estimates of the patient’s state at each observation period. This procedure accounts for the inherent process and measurement noise to provide information to the decision makers that better reflects the actual disease state of each patient.

### Generalized estimating equations

We next used GEE for logistic regression to develop the probability function for significant OAG progression. GEE is an extension of generalized linear models to repeated measures data analysis using quasi-likelihood estimation [23]. GEE is a semiparametric regression technique that uses an iterative algorithm, Newton-Rhapsody, to estimate the coefficient parameters. Unlike linear mixed effects models, GEE is robust to the specification of the correlation structure and requires only the correct specification of the marginal means to obtain consistent and asymptotically normal parameter estimates [24].

Let *y*_*i*,*j*_ be the response (i.e. progression label) for patient *i* at time *j*, and let *μ*_*i*,*j*_ be the expected value of *y*_*i*,*j*_. The GEE approach assumes independence at the patient-level and relates the marginal response, *μ*_*i*,*j*_, to a linear combination of the covariates, *x*_*i*,*j*_, by the link function, *g*(·).

$$\begin{array}{l} g(\mu_{i,j})={\text{x}}_{i,j}^{T}\beta \end{array}$$

where *β* is a *p* × 1 vector of unknown regression coefficients.

Traditionally, the covariates used in GEE are obtained from the raw observed measurements *z*_*i*,*j*_. Our proposed methodology called for using the state estimates ${\hat{\alpha }}_{i,j}$ as the input to our model. We compared the performance of the GEE model which uses ${\hat{\alpha }}_{i,j}$ (Kalman filter estimates) against the GEE model which uses *z*_*i*,*j*_ (raw measurements).

Through the GEE framework, we considered the variance of the response variable, *V*(*y*_*i*,*j*_), to be a function, *v*(·), of the mean response, *μ*_*i*,*j*_:

$$\begin{array}{l} V(y_{i,j})=v(\mu_{i,j})\phi \end{array}$$

where *v*(·) is a known variance function and *ϕ* is a possibly unknown scale parameter.

Because of our Bernoulli response variable, i.e. 1 for progression and 0 for nonprogression, we used the logit link function, logit variance function, and scale parameter, *ϕ* = 1:

$$\begin{array}{l} g(\mu_{i,j})=\text{log}[\;\frac{\mu_{i,j}}{1-\mu_{i,j}}]\\ v(\mu_{i,j})=\mu_{i,j}(1-\mu_{i,j}) \end{array}$$

Repeated measures data are inherently correlated, with independence at the patient level. GEE uses a *n* × *n* “working” correlation matrix, **Z**_*i*_, for each patient’s sequence of response variables **y**_*i*_, to account for this inherent correlation. We utilized an autoregressive correlation structure. The autoregressive correlation structure assumes a first-order relationship between the measurements. The correlation depends on the magnitude of the time difference between the measurements:

$$\begin{array}{l} \text{Corr}(y_{i,s},y_{i,t})=\rho^{\left|s-t\right|},\rho \in (-1,1) \end{array}$$

The final covariate set for the logistic regression was obtained via forward variable selection. Variable selection was initialized with MD, PSD, and change in MD. Chi-squared tests were used to evaluate the benefit of adding a single variable to the model. We iteratively added the variable with the smallest Chi-square test p-value to the model until no new variables were statistically significant (*α*=0.10).

### Model performance

To assess the performance of the logistic regression models, we developed receiver operating characteristic (ROC) curves. 10-fold cross validation was performed to calculate sensitivity and specificities at various discrimination thresholds. Receiver operating characteristic (ROC) curves were created using the average sensitivities and specifities across the 10-fold cross validation to compare the performance of the logistic regression model with raw observations as input versus the model with Kalman filter state estimates as input. Estimates of the area under the ROC curve (AUC) were obtained for each iteration of the 10-fold cross validation.

## Results

The mean (standard deviation) number of visits for patients who met the inclusion criteria was 15.1 (2.6) visits. Nearly 99% of the patients had at least 8 follow-up visits.

We calculated the overall patient average (and standard deviation) of every variable (Kalman filter estimates and raw observations) from the VF and IOP tests (Additional file 1: Table S1) for instances of progression and nonprogression separately. We note that generally the difference between the progressing and nonprogressing means for a variable is larger for the Kalman filter estimates than in the raw measurement data set, e.g. the difference between progressing and nonprogressing PSD is 7.643 for the Kalman filter estimates and 4.831 for the raw measurements. The increased difference between progressing and nonprogressing means for a variable in the Kalman filter data set is due to the linear system dynamics framework of the Kalman filter. As time increases, the linear trajectory of the Kalman filter results in more disparate variable values between nonprogressing and progressing instances compared to the “noisy” trajectories in the raw measurements data set.

We can also see that the standard deviation of the mean of the variables is greater in the Kalman filter estimates than the raw measurements, e.g. the standard deviation of the mean MD of progressing Kalman filter estimates is 6.229 compared to 3.688 for the raw measurements. The higher standard deviation of the mean of the variables for the Kalman filter estimates shows that the Kalman filter sends patients on different trajectories, i.e. each patient has a different average variable value for his/her progressing instances. In the raw observations data set, each patient follows a more similar trajectory, i.e. each patient has a more similar average variable value for his/her progressing instances. In the raw observations data set, each patient’s true trajectory is muddled by process and measurement noise, which results in similar looking trajectories. The Kalman filter, however, reduces noise to extract the true signal which results in trajectories that reflect the patient’s particular disease characteristics.

The final logistic regression models are summarized in Additional file 2: Table S2. Both models use the same set of final variables, however the odds ratios are larger for the model trained on Kalman filter estimates than the odds ratios for the model trained on raw observations. For instance, the odds ratio of PSD is 1.344 for the logistic regression model based on Kalman filter estimates and 1.107 for the logistic regression model based on raw observations. Analysis of the logistic regression fitted values, i.e. the estimated probability of progression, is presented in Table 1. It is important to note that the average estimated probability of progression of the Kalman filter progressing instances (0.738) is much higher than the average for the raw observations progressing instances (0.498). Additionally, the difference between average fitted values of progressing and nonprogressing instances is much greater for the Kalman filter estimates (95% CI 0.611,0.676) than for the raw observations data set (95% CI 0.290,0.337). This increased difference of average fitted values between progressing and nonprogressing instances supports our hypothesis that Kalman filtered estimates allows for improved distinction of significant disease progression.

**Table 1:** Fitted probabilities from logistic regression models

	**Progressing**	**Nonprogressing**	**95% CI**
	**mean (SD)**	**mean (SD)**	**of diff**
Kalman filter estimates	0.738 (0.279)	0.095 (0.172)	(0.611,0.676)
Raw observations	0.498 (0.206)	0.185 (0.111)	(0.290,0.337)

The table provides the mean and standard deviation of the fitted probabilities for progression and nonprogression instances from both logistic regression models.

**Figure 1:**
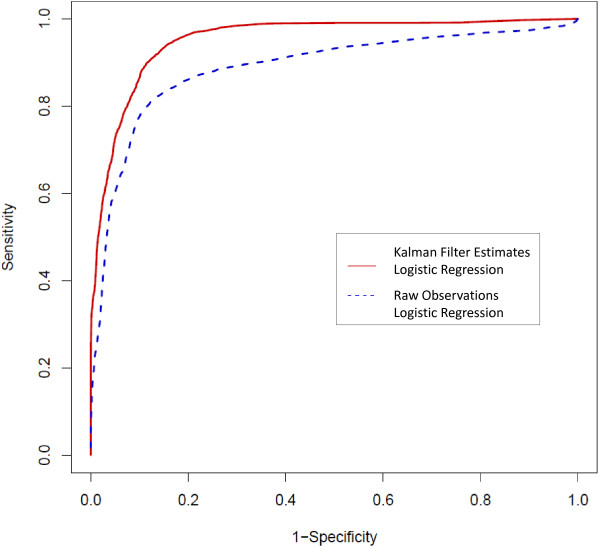
**ROC curves for Kalman filter estimates and raw measurements logistic regression models.** Estimates of sensitivity and specificity obtained via 10-fold cross validation are used to generate the receiver operating characteristic (ROC) curve for the two logistic regression models parameterized with Kalman filter estimates and raw observations.

Finally, the ROC curve, Figure 1, illustrates that by first filtering the data using the Kalman filter model, we can achieve higher sensitivity and specificity than a model based on the raw observations. For example, if we select 95% sensitivity as our goal, we can obtain 83% specificity using the Kalman filter model but only 39% specificity using the raw observations model. At 90% sensitivity, the Kalman filter achieves 88% specificity while the raw observations model achieves 66% specificity. The mean (and variance) of the estimated AUC for the Kalman filter and raw observations models are 0.961 (0.002) and 0.889 (0.013), respectively. Hence, using the probability function generated via Kalman filter state estimates and GEE for logistic regression, we are able to more accurately classify patients and instances as experiencing or not experiencing significant OAG progression.

### Discussion

Using Kalman filter forecasts in determinations of when patients with OAG should be observed by their physician required the development of a mapping from the filtered health metrics to a probability of progression. The application of GEE for logistic regression on Kalman filtered longitudinal observations of patients with OAG resulted in improved ability to identify significant glaucoma progression as compared to the model generated using the raw clinical trial data. The Kalman filter model is able to better detect relationships between health metrics and the more complex disease progression definition than the logistic regression model using raw observations as inputs. We believe that as the progression definition becomes more heavily influenced by systematic process and measurement noise, a logistic regression model parameterized on Kalman filter estimates of the input will become increasingly more beneficial for detecting disease progression.

The methodology we present here takes advantage of state estimation and the linear system model of the Kalman filter, in conjunction with the marginal response of GEE, to improve the logistic regression model’s ability to correctly classify patients. The Kalman filter model performs at higher specificity and sensitivities for significant disease progression classification due to the greater difference in mean fitted values (i.e. average estimated probability of progression) between progressing and nonprogressing instances. As we iterate through potential probability thresholds for classifying instances/patients as progressing or nonprogressing, the greater difference in mean fitted values creates a larger set of thresholds for which there are fewer false negatives and false positives. The lower rate of false negatives and false positives leads to improved sensitivity and specificity for the Kalman filter model at detecting significant glaucoma progression in comparison to the raw measurements.

The difference of mean fitted values for the Kalman filter model is larger because of the greater in magnitude covariate coefficients and higher odds ratios of the model covariates. With higher odds ratios, each unit increase in a predictive covariate increases the probability of progression more greatly for the Kalman filter model than it does for the raw measurements model.

The greater in magnitude covariate coefficients and higher odds ratios are explained by the linear system model the Kalman filter uses for state estimation. In the case of glaucoma, IOP decreases over time for treated patients and mean deviation becomes more negative since VF loss cannot be reversed. The trend creates the larger difference in mean variable values as the number of measurements increases. The “noisy” nature of the raw measurements creates fluctuation around this expected trend. Because the GEE approach is concerned with population-averaged (i.e. the mean response) variable, we expect covariate coefficients to be greater in magnitude when the difference between the mean variable value for progression and nonprogression instances increases.

The increased standard deviation of the mean value of the Kalman filter estimates is due to the Kalman filter’s recognizing each patient’s individual disease realization. As the Kalman filter updates the state estimates to reflect a patient’s particular characteristics, that patient’s trajectory becomes more dissimilar to the trajectories of other patients. The “noisy” raw measurements mask these dissimilar trajectories which results in clustered mean variable values for progressing or nonprogressing instances.

Increased sensitivity and specificity of classification models improves clinical decision making by more accurately identifying significant disease progression. Clinicians who are able to correctly identify patients who experience significant glaucoma progression can make more informed decisions, such as improving monitoring schedules and improving treatment decisions. Additionally, the increased accuracy allows clinicians to utilize the statistical model without fearing high rates of misclassification.

Our proposed methodology is limited by the linear system dynamics model. Glaucoma progresses relatively slowly, thus changes in disease state can be estimated well by a linear model. For more rapidly progressing diseases, if the time between patient observations is sufficiently small, the disease progression mechanics can potentially be estimated by a linear dynamics model. The Kalman filter also assumes the state estimates, noise and raw observations come from a Gaussian distribution. This assumption is reasonable within a range around the mean (2 standard deviations) for bounded variables, e.g. IOP.

The application of our methodology to CIGTS data is limited by the fact that this trial took place between 1993 and 2003. Since 2003, there have been many advances in the field of diagnostic testing for glaucoma including testing to check for damage to the retinal nerve fiber layer tissue using optical coherence tomography (OCT) and additional progression detection software on the visual field machines such as Guided Progression Analysis (GPA). In the future, we plan to use data from other sources to be able to integrate data from OCT and GPA into our models and progression definition.

## Conclusion

In this paper, we applied a linear system dynamics model approach, using a Kalman filter, to estimate true measurement values for variables which have both measurement and process noise. Filtering techniques are important for true measurement estimation for medical decision making and have been shown to result in improved significant disease progression classification when utilizing GEE for logistic regression with repeated measures data, as demonstrated in our modeling of OAG progression dynamics. Due to process and measurement noise, only after having seen future observations can clinicians retrospectively assess whether “true” progression has occurred. Logistic regression models that directly consider those noises allow for the prospective calculation of the probability of experiencing progression. Furthermore, for complex progression definitions, logistic regression enables a reduction in the number of variables to consider, which is important in guiding clinical decisions. This methodology is also applicable to other chronic diseases, particularly those diseases whose dynamics can be modeled effectively by a linear system and whose biomarkers can be reasonably approximated by a Gaussian distribution.

## Competing interests

All authors currently have a provisional patent and have filed a patent application (“Patient-Specific Modeling and Forecasting of Disease Progression”, Atty. Docket No. 010109-11001A) related to the content of this manuscript.

## Authors’ contributions

GS prepared the manuscript, assisted in the design of the study, and performed the computational analysis. ML conceived and supervised the research, provided intellectual guidance, and revised the manuscript. JS provided subject matter expertise, supervised analysis, and revised the manuscript. DM provided the clinical trial data and subject matter expertise. All authors read and approved the final manuscript.

## Pre-publication history

The pre-publication history for this paper can be accessed here:

http://www.biomedcentral.com/1472-6947/13/137/prepub

## Supplementary Material

Additional file 1 **Summary of Kalman filter estimates and raw observations.** The table provides the overall mean and standard deviation for the variables at progressing and nonprogressing instances. Kalman filter estimates and raw observations of these variables are compared. Click here for file

Additional file 2 **Coefficients of logistic regression.** The table presents the final covariate sets for the two logistic regression models parameterized with Kalman filter estimates and raw observations. We present the mean (variance) of the coefficient parameters of the iterations of the 10-fold cross validation. Click here for file
