# Supplementary material for: Filtering data from the collaborative initial glaucoma treatment study for improved identification of glaucoma progression
Source: BMC Med Inform Decis Mak. 2013 Dec 21;13:137. doi: 10.1186/1472-6947-13-137 (PMC3878032; doi:10.1186/1472-6947-13-137)
Supplement: Additional file 1 — Summary of Kalman filter estimates and raw observations. The table provides the overall mean and standard deviation for the variables at progressing and nonprogressing instances. Kalman filter estimates and raw observations of these variables are compared. [file 1472-6947-13-137-S1.pdf]

	Kalman Filter Estimates		Raw Measurements	
Variable	Progressing Mean (SD)	Nonprogressing Mean (SD)	Progressing Mean (SD)	Nonprogressing Mean (SD)
MD	-8.75 (7.38)	1.24 (4.79)	-8.55 (4.30)	-2.31 (2.54)
MD Velocity	1.94 (3.78)	2.37 (2.50)	-.41 (1.43)	0.01 (0.94)
MD Acceleration	2.44 (3.78)	2.31 (2.540)	-.04 (1.14)	-0.01 (0.85)
IOP	18.01 (5.30)	19.25 (5.14)	17.47 (3.82)	17.62 (3.10)
IOP Velocity	2.50 (3.87)	2.48 (3.02)	-.03 (2.00)	-0.10 (1.64)
IOP Acceleration	2.42 (3.56)	2.42 (2.74)	.05 (1.67)	0.01 (1.49)
PSD	12.57 (6.31)	4.93 (4.03)	8.20 (3.45)	3.37 (2.17)
Baseline MD	-5.35 (3.71)	-3.15 (2.60)	-5.35 (3.71)	-3.15 (2.60)
Baseline IOP	28.070 (5.70)	27.69 (5.04)	28.07 (5.70)	27.69 (5.04)
MD Change	-3.41 (6.56)	4.39 (3.52)	-3.21 (4.00)	0.84 (1.85)
IOP Change	-10.06 (7.53)	-8.44 (7.10)	-10.60 (5.21)	-10.07 (4.49)
