# Supplementary material for: Filtering data from the collaborative initial glaucoma treatment study for improved identification of glaucoma progression
Source: BMC Med Inform Decis Mak. 2013 Dec 21;13:137. doi: 10.1186/1472-6947-13-137 (PMC3878032; doi:10.1186/1472-6947-13-137)
Supplement: Additional file 2 — Coefficients of logistic regression. The table presents the final covariate sets for the two logistic regression models parameterized with Kalman filter estimates and raw observations. We present the mean (variance) of the coefficient parameters of the iterations of the 10-fold cross validation. [file 1472-6947-13-137-S2.pdf]

	Kalman Filter Estimates			Raw Observations		
Variable	Mean (Variance) of Coefficient	Odds Ratio	P Value	Mean (Variance) of Coefficient	Odds Ratio	P Value
Intercept	-3.492 (0.104)	0.030	<0.001	-1.966 (0.019)	0.140	<0.001
MD	-0.554 (0.002)	0.575	<0.001	-0.178 (0.000)	0.837	<0.001
MD Velocity	0.193 (0.001)	1.213	0.06	0.071 (0.000)	1.074	0.04
Baseline MD	0.813 (0.004)	2.256	<0.001	-0.009 (0.001)	0.991	0.86
PSD	0.297 (0.000)	1.345	<0.001	0.102 (0.000)	1.108	0.005
IOP	0.076 (0.000)	1.079	0.03	-0.024 (0.000)	0.976	0.03
